# Supplementary material for: Combined PTPN11 and MYBPC3 Gene Mutations in an Adult Patient with Noonan Syndrome and Hypertrophic Cardiomyopathy
Source: Genes (Basel). 2020 Aug 17;11(8):947. doi: 10.3390/genes11080947 (PMC7463848; doi:10.3390/genes11080947)
Supplement: Supplementary file 1 [file genes-11-00947-s001.pdf]

Supplementary Table 1. List of the 325 genes included in the NGS panel.

Gene	Read Depth	Mean Coverage
A2ML1	333.29	96.77
ABCA1	304.22	97.83
ABCB1	295.88	95.83
ABCC9	272.36	97.44
ABCG5	383.73	100
ABCG8	346.5	100
ACE	330.74	100
ACTA2	269.88	100
ACTC1	414.6	100
ACTN2	230.54	100
ADAMTS10	459.57	92.86
ADAMTS9	300.97	100
ADAMTSL4	1362	80
ADCY5	313.91	91.3
ADD1	348.93	100
ADRA2A	660	100
ADRB1	394.33	100
ADRB2	1313	100
AGT	562.5	100
AGTR1	1288	100
AKAP9	388.61	90.2
ANK1	299.86	97.62
ANK2	422.7	82.61
ANKRD1	303.86	100
APOA1	684.5	100
APOA2	271.67	100
APOA5	816	100
APOB	675.52	96
APOC2	185.33	100
APOC3	387.5	100
APOE	365.25	100
APP	255	94.44
ARHGAP31	461.62	92.31
ARL6	282.86	85.71
ATP11A	279.03	96.55
B2M	226	100
B3GAT3	472.5	100
BAG3	563.5	100
BBIP1	232.5	100
BBS1	318.29	92.86
BBS10	1271.5	100
BBS12	2253	100
BBS2	346.33	93.33

BBS4	246.4	100
BBS5	287	80
BBS7	284	94.12
BBS9	270.1	95.24
BCL11A	808	75
BRAF	254.94	88.89
C2CD4B	352	100
C8orf37	256.33	83.33
CACNA1C	298.44	100
CACNA2D1	234.39	81.58
CACNB2	283.47	94.74
CALM1	311	100
CALM2	278.33	100
CAMK1D	269.55	90.91
CASQ2	263.73	81.82
CAV3	364	100
CBS	306.31	100
CCDC115	303	100
CCND1	306.8	100
CDC123	222.15	92.31
CDKAL1	274	93.33
CDKN2A	316.2	100
CDKN2B	393.5	100
CEP290	282.36	96
CETP	343	61.54
CHD7	415.94	97.06
CITED2	1232	100
CLCNKB	325.6	93.33
COL1A2	289.03	91.89
COL3A1	355.29	79.41
CREB3L3	423.75	100
CRY2	315	100
CRYAB	445	100
CSRP3	277.2	100
CST3	230.67	100
CTNNA3	248	100
CUL3	275.19	93.75
CYP11B1	555.17	100
CYP11B2	495.57	100
CYP27A1	521.8	100
CYP2C19	364	100
CYP2C9	364.5	100
CYP2D6	487.83	100
CYP3A4	300.77	100
CYP7A1	379.67	100
DES	551.4	100

DGKB	258.83	91.67
DHCR7	355.71	100
DLL4	365.22	100
DMD	249.83	100
DOCK6	392.73	93.94
DOLK	1731	100
DSC2	340.13	93.33
DSG2	412.92	100
DSP	517.92	100
DTNA	282.28	88
DUSP9	369	100
DYNC2LI1	231.08	100
ELN	363.46	84.62
EOGT	216.79	100
ESR1	357.62	100
EVC	342.25	100
EVC2	324.68	95.45
F2	395.4	100
F5	383.16	100
F8	424.81	92.59
FADS1	357.89	100
FBN1	298.53	98.33
FBN2	262.77	95.31
FGA	572.33	100
FGB	388.43	100
FGG	363.75	87.5
FHL2	333.2	100
FKTN	274.22	100
FLNC	541.96	100
FN3K	324.67	100
FOXE3	282	100
FTO	312.44	88.89
G6PC2	327.6	100
GATA4	298.67	83.33
GATA5	343.67	100
GATA6	371.83	100
GATAD1	247.2	100
GCK	369.8	90
GCKR	278.75	87.5
GDF1	252.25	100
GIPR	298	100
GJA5	1330	100
GLI1	417.56	100
GLIS3	417.45	100
GNAS	667.8	100
GPD1L	284.12	87.5

GPIHBP1	409.33	100
GSN	287.79	94.74
HCN4	666.71	100
HFE	428.6	100
HHEX	402	100
HK1	301.68	90.91
HMGA2	214	100
HMGCR	362.07	100
HNF1A	515	87.5
HNF1B	276.67	100
HRAS	446.33	100
IDE	244.33	96.3
IFT172	305.32	100
IFT27	289.29	100
IGF1	266.67	83.33
IGF2BP2	257.06	100
IL1B	279.33	83.33
IL1RN	247.17	83.33
INS	361	100
INSR	379.05	100
ITGB3	294.14	85.71
ITM2B	284.33	83.33
JAG1	380	95.24
JAZF1	336.8	100
JPH2	432.71	100
JUP	396.25	100
KAT6B	520.25	93.75
KCNA5	1955	0
KCND3	430.14	100
KCNE1	615	100
KCNE2	489	100
KCNE3	450	100
KCNH2	397.53	100
KCNJ11	1454	100
KCNJ2	1422	100
KCNJ5	772.5	100
KCNJ8	794	100
KCNQ1	280.06	100
KDM6A	284.27	100
KIF11	295.71	100
KLF14	773	100
KLHL3	256.27	100
KMT2D	1037.22	82.61
KRAS	263.4	80
LAMA4	269.95	100
LCAT	707.67	100

LDB3	379.12	87.5
LDLR	363.62	100
LDLRAP1	373.25	75
LIG4	2889	100
LIPA	286.44	88.89
LIPC	322.5	90
LIPI	267.1	90
LMF1	340	100
LMNA	435.45	100
LOX	306.29	85.71
LPL	361.9	90
LTBP2	340.91	96.97
LYZ	245.25	100
LZTFL1	271.64	90.91
LZTR1	178.16	100
MADD	379.18	92.86
MAP2K1	284.82	100
MAP2K2	297.9	90
MFAP5	263	87.5
MIB1	300.62	100
MIB2	339.91	100
MKKS	580.25	50
MKS1	312.87	100
MTHFR	402.09	90.91
MTNR1B	626.5	100
MTTP	268.94	100
MYBPC3	349.09	95.65
MYH11	333.06	91.67
MYH6	382.9	100
MYH7	419.59	100
MYL2	252.5	100
MYL3	356.75	100
MYL4	211	100
MYLK	318.87	93.55
MYPN	298.67	95.24
NAT2	991	100
NEBL	251.29	93.55
NEXN	387.1	90
NKX2-5	810	100
NOS1AP	313.9	100
NOS3	407.63	94.74
NOTCH1	458.39	89.29
NOTCH2	380.15	93.94
NPHP1	240.1	100
NPPA	553	100
NR3C2	505	100

NRAS	269.25	100
NUP155	248.53	100
OFD1	295.3	95
OSMR	318.18	100
PCSK9	501.7	90
PITX2	444.6	80
PKP2	347.92	92.31
PLEKHM2	179.17	100
PLN	253	100
PLOD1	311.68	89.47
PPARG	370	100
PRC1	312.21	100
PRDM16	303.88	100
PRKAG2	286	100
PRKG1	266.61	94.44
PROX1	669	100
PSEN1	296.6	100
PSEN2	277.2	90
PTPN11	311.29	100
RAF1	336.69	100
RANGRF	407.33	100
RAP1A	196.5	100
RAP1B	165.17	100
RASA2	229.83	100
RBM20	395.64	100
RBPJ	387	45.45
RIT1	338.75	100
RRAS	446	100
RTN1	386.89	100
RYR2	272.37	97.14
SAA1	308	100
SAR1B	233.67	83.33
SCAP	300.35	100
SCN10A	352.73	96.15
SCN1B	426.75	100
SCN2B	329.5	75
SCN3B	285.6	100
SCN4B	273.4	100
SCN5A	352.22	100
SCNN1B	372	90.91
SCNN1G	418.4	100
SDCCAG8	304.88	100
SDHA	370.31	100
SEMA3E	296.12	70.59
SGCD	253.88	100
SHOC2	357.38	100

SKI	528.33	100
SLC12A1	292.3	96.3
SLC12A3	353.17	87.5
SLC2A10	565.25	100
SLC2A2	299.1	100
SLC30A8	287.62	100
SLMAP	238.47	100
SMAD3	317.11	100
SNTA1	388	100
SOS1	309.36	100
SOS2	333.14	90.91
SPTA1	264.63	95.92
TAF1A	270.5	100
TAZ	398.5	100
TBX5	350.38	100
TCAP	384.5	100
TCF7L2	295.83	100
TGFB2	352.14	100
TGFB3	291	100
TGFBR1	306.62	100
TGFBR2	338.25	87.5
THADA	286.56	100
TMEM199	280.2	100
TMEM43	268.75	100
TMPO	470.89	100
TMPRSS6	317.18	100
TNF	399.33	100
TNNC1	205.2	100
TNNI3	291.57	100
TNNT2	283.29	78.57
TNXB	597	100
TP53INP1	332.75	100
TPM1	281.71	100
TRDN	216.25	80
TRIM32	2060	100
TRPM4	392.17	100
TSPAN8	216.57	100
TTC8	269	100
TTN	930.77	94.08
TTR	265.25	75
TUBGCP3	295.27	90.91
TXNRD2	310.81	87.5
USF1	542.17	100
VCL	276.05	100
VPS13C	273.86	96.39
WDPCP	292.76	82.35

WFS1	564.57	100
WNK1	413.04	100
WNK4	539.36	100
ZBED3	207	100
ZFAND6	296.8	100
ZFPM2	571.38	100
